# Narrative Review on Vestibular Complaints After Cochlear Implantation in Adults: Defining Heterogeneous Common Symptoms

**DOI:** 10.3390/audiolres16020050

**Published:** 2026-03-25

**Authors:** Francesco Lazzerini, Francesca Forli, Stefano Berrettini, Federica Di Berardino, Marco Pozzi, Diego Zanetti

**Affiliations:** 1 Otolaryngology, Audiology, and Phoniatrics Unit, University of Pisa, 56127 Pisa, Italy; francesca.forli@gmail.com (F.F.); s.berrettini@med.unipi.it (S.B.); 2 Hearing Implant Section, Karolinska Institutet, 171 77 Stockholm, Sweden; 3 Audiology Department, Fondazione IRCCS Cà Granda Ospedale Maggiore Policlinico di Milano, 20122 Milan, Italy; federica.diberardino@unimi.it (F.D.B.); marco.pozzi@policlinico.mi.it (M.P.); diego.zanetti@unimi.it (D.Z.); 4 Dipartimento di Scienze Cliniche e di Comunità, Università degli Studi di Milano Statale, 20122 Milan, Italy

**Keywords:** cochlear implantation, vertigo, vestibular dysfunction, benign paroxysmal positional vertigo, endolymphatic hydrops

## Abstract

Cochlear implantation (CI) effectively restores hearing across the whole lifespan but may be followed by vestibular complaints, especially in adult recipients. The aim of this narrative review is to provide a comprehensive characterization of vestibular complaints after CI in adults, collecting clinical and instrumental data, as well as discussing the risk factors for their development. From data reported in the literature, we defined five recurring clinical presentations of postoperative vestibular disturbances (phenotypes): acute postoperative vestibular syndrome, benign paroxysmal positional vertigo (BPPV), delayed Ménière-like vertigo attributable to secondary endolymphatic hydrops, chronic postoperative disequilibrium, and stimulation-linked vertigo. According to the different pathogeneses underlying each presentation, the management of postoperative vestibular complaints should be phenotype-guided, including short-course vestibular suppressants and early mobilisation for acute presentations; canalith repositioning for BPPV; empiric therapy for hydropic-like episodes; and vestibular rehabilitation when imbalance is persistent, programming changes for stimulation-linked symptoms. Alongside this phenotype-driven approach, subjective symptoms are common across cohorts but are usually transient and persistent disability is uncommon. Furthermore, instrumental data across the studies indicate that objective abnormalities cluster in otolith and low-frequency canal measures: Cervical, ocular VEMP, and caloric responses are more often impaired than high-frequency canal function on vHIT, confirming histopathological studies showing preferential saccular involvement during the insertion of the electrode array. The risk of postoperative vestibular complaints not only appears to be modulated more by patient-related factors, especially pre-existing vestibular loss, but also by the aetiology of deafness, or age, rather than by device characteristics; atraumatic surgical approaches may further reduce this risk. This review emphasizes that future research on vestibular complaints after CI should adopt standardized phenotypes when evaluating symptoms, objective vestibular function, falls, and quality of life. Additionally, it should correlate these outcomes with hypothetical risk factors and detailed surgical reports.

## 1. Introduction

Cochlear implantation (CI) is nowadays considered a highly effective intervention for the auditory rehabilitation of hearing-impaired individuals across all ages [1,2,3], with well-documented benefits in terms of speech and hearing abilities, as well as quality of life (QoL). One of the most frequently reported complications following CI is the occurrence of transitory balance disorders [4,5,6].

Approximately 30–40% of CI recipients complain of vestibular symptoms, most often transient and typically presenting as delayed episodic vertigo or postural disequilibrium [7,8,9]. The literature shows a large heterogeneity in the definition of vestibular symptoms, in the diagnostic protocols adopted, and in the reported incidence rates, with studies relying on a wide variety of subjective and objective vestibular assessments and follow-up durations [10,11,12].

Acute postoperative symptoms typically arise within days to weeks after surgery and include spontaneous rotatory vertigo with postural imbalance, which in most cases resolve within a few days or weeks [8,13,14,15,16]. Additionally, benign paroxysmal positional vertigo (BPPV)-like episodes have been reported, presenting with typical or atypical positional vertigo [8,14,17,18,19].

Delayed, late-onset, or chronic symptoms, occurring months to years after implantation, may present as Ménière-like episodic vertigo or fluctuating imbalance and likely reflect chronic inner ear changes such as endolymphatic hydrops or fibrosis [7,8,14,17,18,20]. Moreover, persistent disequilibrium has also been reported and appears more common in patients with pre-existing vestibular impairment or bilateral involvement [8,14,17,18,20].

In addition, anecdotic cases of electrically induced vestibular disturbances such as oscillopsia, visual tilt, or lateropulsion have been described [8,14,17].

Taken together, these observations underline the heterogeneous nature of vestibular disorders following CI, ranging from transient postoperative dizziness to persistent imbalance or delayed hydropic-like crises, with significant variability in onset, risk factors, presentation, and prognosis.

The aim of this review is to provide a comprehensive characterization of the different clinical phenotypes of vestibular dysfunction in cochlear implant recipients, summarizing the main findings from the literature and reporting practical implications for diagnosis, management, and counselling.

## 2. Methods

This is a narrative review conducted with a transparent approach to the literature. As a narrative review, it did not follow a full systematic review protocol. We performed structured searches in PubMed and Scopus using combinations of the following terms related to cochlear implantation and vestibular symptoms: cochlear implant, vertigo, dizziness, imbalance, benign paroxysmal positional vertigo, endolymphatic hydrops, and vestibular tests. Approximately 250 records were retrieved. Titles and abstracts were screened to identify studies reporting postoperative vestibular symptoms, objective vestibular assessments, or pathophysiological mechanisms. Non-adult populations, animal studies, surgical technique papers without vestibular outcomes, and articles not written in English were excluded, resulting in roughly 100 papers considered potentially relevant. After full-text assessment, we retained around 80 articles that directly addressed vestibular symptoms after cochlear implantation or provided instrumental or histopathological evidence relevant to postoperative vestibular function.

### 2.1. Methodological Limitations

A narrative approach was intentionally adopted to provide a clinically oriented synthesis of the highly heterogeneous literature. Unlike systematic reviews, the study selection process was not based on fully reproducible criteria, and no formal risk-of-bias assessment or quantitative synthesis was performed. Consequently, the possibility of selection bias cannot be entirely excluded, and the strength of the evidence should be interpreted with caution. No causal inferences or independent risk estimates can be derived; therefore, associations discussed in the literature (e.g., age or aetiology) should be interpreted as descriptive trends rather than definitive predictors.

Within this context, the primary aim of this review was not to generate pooled estimates but rather to propose a phenotype-based framework capable of supporting clinical reasoning, patient counselling, and future research standardization.

Future studies adopting standardized definitions and prospective designs will be essential to enable higher-level evidence synthesis.

Main studies considered in the present review were reported in Table S1, in supplementary files.

### 2.2. Disclosure

Generative AI tools were used to assist with text generation; study design; literature searching/data collection; and grammar, spelling, and punctuation checks.

## 3. Results

Across the heterogeneous literature, five recurring clinical phenotypes of vestibular complaints following cochlear implantation can be identified.

The first phenotype comprises acute postoperative vestibular symptoms occurring within the first month after surgery and typically resembling an acute unilateral vestibular deficit. The second phenotype is represented by benign paroxysmal positional vertigo (BPPV)-like episodes. The third includes delayed Ménière-like symptoms, likely related to secondary endolymphatic hydrops. The fourth phenotype consists of chronic postoperative imbalance, often reflecting an exacerbation of pre-existing vestibular impairment or incomplete central compensation. Finally, a fifth and rare phenotype is related to electrically induced vestibular symptoms triggered by device activation.

The following sections provide a detailed synthesis of the available literature for each phenotype. On this phenotype-based framework, several modifying factors—such as patient-related characteristics and surgical determinants—may influence clinical expression and outcomes; these aspects are addressed in Section 4.

### 3.1. Acute Postoperative Vertigo

In our review of the literature, we defined acute postoperative vertigo as non-positional rotatory vertigo and/or acute imbalance occurring within the first 30 days after surgery, with either an immediate (0–72 h) or early presentation (4–30 days), in line with previous classifications distinguishing early, prolonged, and delayed vertigo after cochlear implantation from Ito, 1998 [21]. This clinical picture excludes purely positional vertigo, which will be discussed further as a separate manifestation.

The reported incidence ranges between 22% and 45% in several cohorts of patients with acute vestibular symptoms in the immediate postoperative period [8,22,23]. Most cases are transient, resolving within days to weeks; persistence at 4 weeks is uncommon (<5%), and long-term sequelae are rare [13,22].

Pathogenesis may be related to the acute unilateral hypofunction from surgical trauma, as well as to transient labyrinthine inflammatory reaction, and to perilymphatic disturbances such as fistulae or pneumolabyrinth [8,24].

Bedside assessments usually document spontaneous nystagmus, “beating” towards the non-operated side in the case of an acute vestibular hypofunction from surgical trauma, or towards the operated side, in the case of labyrinthine irritation.

Instrumental evaluations with video Head Impulse Test (vHIT), caloric stimulation, cervical vestibular evoked myogenic potentials (cVEMP), and ocular VEMP (oVEMP) could help quantify the semicircular canals and the otolithic involvement. In fact, several studies suggest that surgical trauma from CI can produce some degree of injury to the ipsilateral labyrinth, causing clinically relevant symptoms. This aspect is discussed further below.

Also, histopathologic studies reported significant damage of vestibular structures in nearly 55% of specimens, with the saccule most frequently affected [25], a pattern consistent with the high rate of cVEMP abnormalities observed in several clinical cohorts [22,26].

Despite all the potential harm to the labyrinth function, paradoxically, device activation has been reported to improve balance performance in most recipients, possibly by enhancing multisensory integration and facilitating central compensation [27].

Management is primarily supportive, with short courses of vestibular suppressants, antiemetics, and early mobilisation, as symptoms usually rapidly stabilize. Corticosteroids may be considered in “irritative” phenotypes, though evidence is limited and heterogeneous [9,28].

When a perilymphatic fistula is suspected (with associated sound- or pressure-induced vertigo or nystagmus elicited by Valsalva or tragal pressure), conservative measures are preferred, reserving surgical exploration for refractory cases. In the latter instance, an imaging assessment with high-resolution or ultra-high-resolution CT may be indicated [24,29].

As discussed further below, associations between acute postoperative vestibular syndrome risk and surgical approach (round-window vs. cochleostomy), electrode type, or insertion depth remain inconsistent, in addition to patients’ age or aetiology of hearing loss. Generalizable conclusions about these factors are limited by methodological heterogeneity [28,30,31,32].

### 3.2. Postoperative BPPV-like Vertigo

BPPV after cochlear implantation is uncommon but consistently reported in adult cohorts, with rates ranging between 2% and 11% in implanted populations, much higher than the 2–3% estimated in the general adult population [8,19,23,24,33,34].

Time-to-onset varies widely. Most cases arise within the first months, and some episodes occur even before device activation; delayed presentations are also documented [19,33,34].

The posterior canal is affected most frequently (approximately 75–90%), with occasional lateral canal involvement [19,33,34,35,36].

Several mechanisms have been proposed to explain the occurrence of BPPV after CI. The first hypothesis suggests that bone dust particles generated during cochleostomy may enter the cochlea and, through a microdisruption of the basilar membrane, gain access to the endolymphatic space and subsequently the semicircular canal lumen, where they act as canaliths and induce BPPV [34]. A second mechanism considers the mechanical effect of drilling: Vibratory forces applied to the cochlea may be sufficient to detach otoconia from the utricular macula, allowing them to migrate into the semicircular canals and cause canalolithiasis [19,33]. Finally, a third theory, proposed by Di Girolamo et al. (1999), although with less clinical evidence, implicates electrical stimulation during the initial device fitting, whereby current spread or mechanical activation could precipitate otolith displacement [35].

Diagnosis rests on positional testing (Dix–Hallpike, supine roll), with canal-specific nystagmus patterns [19,24,33,34,35,37]. When assessing early postoperatively, head maneuvering should be cautious but is generally feasible.

Treatment echoes the standard practice in non-implanted patients with canalith repositioning maneuvers: Semont or Epley maneuvers for the posterior canal; Gufoni, McClure, or Lempert for the horizontal canal [38].

Most series report the resolution of BPPV after one to two maneuvers and no adverse effects on device function [19,33,34,35].

Recurrence rates appear comparable to idiopathic BPPV; relapses within three months are described and typically respond to repeat maneuvers. Predictors of recurrence, such as age, deafness aetiology, or device type, have not been identified [19,24,33].

### 3.3. Postoperative Ménière-like Delayed Vertigo and Endolymphatic Hydrops

Ménière-like vertigo may occur months to years after CI in adults, presenting with fluctuating vestibular symptoms that sometimes mimic classic Ménière’s disease. Among the studies reviewed, only a few specifically reported hydropic or Ménière-like presentations following cochlear implantation, confirming that the presence of this phenotype is scattered in the literature.

The literature, indeed, presents significant heterogeneity in incidence, timing, clinical presentation, diagnostic approaches, and outcomes, with reported incidence rates of post-CI episodic vertigo attributable to secondary hydrops ranging from 5% to 16% among CI recipients and with onset occurring from several months up to years after implantation [8,22,23,39,40].

Clinical features of postoperative Ménière-like delayed vertigo include episodic vertigo, fluctuating imbalance, aural fullness, hearing distortion (even through CI), and tinnitus, often indistinguishable from primary Ménière’s disease [39,41]. Along with ipsilateral hydropic symptoms, even contralateral endolymphatic hydrops has also been described [41]. In this context, the term “delayed” refers to vestibular symptoms occurring beyond the acute postoperative period (i.e., >30 days after surgery), potentially months or years after cochlear implantation, in line with the classical definition of delayed endolymphatic hydrops.

An additional anecdotical aspect observed by several authors in sporadic recipients experiencing postoperative Ménière-like delayed vertigo is the intermittent fluctuation of CI electrical impedances [41,42].

Many underlying pathophysiological mechanisms of CI-hearing fluctuations remain unclear, but many theories have been proposed. It has been speculated that fluctuations may be attributable to endolymphatic hydrops, especially when implantation is performed while Ménière’s disease is still in an active phase [43,44,45,46]. On the other hand, pathophysiological studies suggest that fibrosis and obstruction of the *ductus reuniens* can lead to combined cochlear and vestibular endolymphatic hydrops due to altered inner ear fluid homeostasis [39,40,41]. An autoimmune response directed against the inner ear in reaction to labyrinthine trauma occurring during CI surgery has been considered as a factor in developing postoperative endolymphatic hydropic conditions [47,48,49].

Diagnosis can rely on standard vestibular testing, while MRI with gadolinium could visualize hydrops in the contralateral ear, and ipsilateral imaging can be limited by implant artifact [41,50]. Mainly, video-oculography during attacks may help characterize ictal nystagmus [39]. Also, vestibular assessments frequently demonstrate fluctuations in amplitude or asymmetric VEMPs [51].

Clinical outcomes are variable, with some patients experiencing long-lasting recurrent or persistent vertigo, while others improve spontaneously over time.

Management strategies include corticosteroids for acute episodes and dietary modifications, although evidence for medical therapy efficacy is limited [39,52].

Theoretical risk factors include surgical technique (cochleostomy vs. round window), electrode type (straight vs. pre-curved arrays), and pre-existing vestibular dysfunction [40], but at present, no strong evidence supports specific features as predictors of postoperative hydropic conditions.

### 3.4. Chronic Postoperative Balance Disorders

Chronic or persistent imbalance is a recognized but heterogeneous complication in adult CI recipients. Objective evidence of vestibular dysfunction is common, yet the clinical relevance of these findings is often limited. In one cross-sectional study, 79% of CI users demonstrated measurable balance impairment and 50% experienced gaze instability, while subjective dizziness scores remained generally low [37]. Gait disturbances, reduced gait speed, and abnormal functional gait assessments were documented, and 42% of participants fell during challenging balance conditions. Nevertheless, most patients did not report disabling vestibular disorders, suggesting that the clinical burden is often modest despite clear subclinical deficits [37].

The main mechanism proposed for chronic postoperative imbalance is bilateral vestibular hypofunction. This may result either from sequential or simultaneous bilateral cochlear implantation (bilateral CI) or from a deterioration of vestibular function on the implanted side superimposed on a pre-existing bilateral vestibular deficit identified preoperatively [28]. However, in most cases, these abnormalities have limited clinical translation, with vHIT, posturography, and patient-reported outcomes often remaining within functional ranges [37]. Clinically, some papers report chronic postoperative imbalances in 2–4.5% of cases [8,22].

In addition, persistent postural-perceptual dizziness (PPPD) can be considered as a potential contributor to chronic disequilibrium in cochlear implant recipients, particularly when symptoms appear disproportionate to objective vestibular deficits [53].

It has also been suggested that age at implantation may be an independent risk factor for postoperative vestibular complaints, particularly chronic imbalance. As discussed later, age should be considered alongside other putative modifiers—pre-existing vestibular impairment, bilateral implantation, and certain surgical techniques—although evidence for their predictive value remains inconsistent [28,31,37].

Diagnostic work-ups for CI recipients with chronic balance disorders typically combine objective vestibular tests (VEMPs, caloric testing, vHIT, and posturography) with subjective measures such as the Dizziness Handicap Inventory (DHI) or Vestibular Rehabilitation Benefit Questionnaire (VRBQ). Across the literature, these approaches underscore a frequent mismatch between objective dysfunction and the relatively mild subjective burden [37].

In terms of functional outcomes, persistent imbalance can reduce confidence in daily activities and modestly increase fall risk. However, many patients adapt well, and the clinical impact is often less severe than expected from test abnormalities. Quality-of-life assessments reflect that equilibrium disturbances contribute only partially to long-term morbidity in CI users [31,37], justifying that management is often mainly conservative, mostly relying on vestibular rehabilitation in symptomatic cases, though many patients require minimal or no intervention [28,37].

### 3.5. Electrically Induced Vestibular Symptoms

Electrically induced vertigo is an uncommon but recognized complication in adult CI users.

Across adult cohorts, only a minor subset of patients’ vestibular complaints are time-locked to sound or electrical stimulation [54,55,56]. Anyway, in a retrospective observational study on their personal series, Coordes and colleagues found that, postoperatively, 18% of adult CI users started to experience sound-induced vertigo using their CI. This phenomenon was usually related to situations with high loudness volumes and broadband noise (e.g., city traffic) [57]. Affected patients described it as increased body sway (35%), spinning/turning (30%), a combination of both sensations (20%), or other vertigo expressions (15%) [57].

Several pathological mechanisms have been proposed. Current spread with cross-stimulation of vestibular end organs (particularly saccular afferents) is considered the leading pathway in stimulation-linked cases [57].

Diagnosis relies on establishing stimulation–symptom coupling. Structured on–off testing during activation or programming supports an electrical aetiology [57]. Objective assessments with video-oculography, caloric irrigation, and VEMPs may document nystagmus or otolith/canal involvement, but evidence guiding their use in this uncommon presentation is scarce.

Management is primarily conservative and focuses on programming adjustments. Selective deactivation of provoking electrodes can abolish symptoms [57]. Modifying stimulation parameters (pulse width or rate, current levels, and, when relevant, grounding/mode) may reduce vestibular co-stimulation; evidence remains limited but clinically pragmatic. In patients where stimulation-induced vertigo coincides with poor auditory performance, postoperative high-resolution or ultra-high-resolution CT may be useful for detecting/excluding electrode misplacement into the vestibular portion of the inner ear [24,29].

Outcomes are generally favorable. Most cases are transient or respond to mapping changes and rehabilitation [57].

## 4. Discussion

In line with the aims of this review, the following discussion reports the available evidence according to the main clinical phenotypes of postoperative vestibular dysfunction, highlighting implications for diagnosis, management, and patient counselling.

As our analysis of the literature indicates, vestibular disturbances after CI are rather common but heterogeneous. Early reports indicate that the incidence of vestibular complaints after cochlear implantation ranges widely, from 0.3% to 75% [57], a variability probably attributable to the inclusion of different clinical presentations, such as nonspecific postoperative dizziness, rotatory vertigo, and BPPV.

The main sources of heterogeneity are the following:

- Symptom definitions, with some authors focusing on self-reported dizziness while others adopt operational clinical diagnoses.
- Timing of assessment, ranging from the immediate postoperative period to long-term follow-up.
- Vestibular test battery used (e.g., caloric testing, vHIT, cVEMP/oVEMP, posturography) pre- and/or postoperatively.
- Follow-up duration, which contributes to variability, since transient disturbances may resolve through central compensation, whereas delayed phenotypes can emerge months or years after implantation.
- Population characteristics, including unilateral versus bilateral implantation and the presence of preoperative vestibular dysfunction.

Against this fragmented background, however, many authors have considered which factors may modulate both risk and clinical expression of post-CI vestibular symptoms. These span patient-related modifiers (age, hearing loss aetiology, and pre- and postoperative state of vestibular system), surgical determinants (approach, insertion trauma, scalar translocation, and perilymphatic events), or device and programming variables (electrode type or positioning, current spread, and electrical parameter settings).

It should be noted that statistically significant associations are inconsistently reported across studies, largely due to heterogeneous designs, small sample sizes, and non-standardized outcome measures; therefore, conclusions regarding risk factors and mechanisms should be interpreted as hypothesis-generating rather than being definitive.

### 4.1. Patient Related-Modifiers

#### 4.1.1. Age

Because vestibular complaints are common in older people [58,59], several authors have examined whether postoperative vestibular complications are more frequent or severe in the elderly, particularly with respect to chronic imbalance. In this regard, Krause et al. (2008) reported a significant association between advanced age and postoperative dizziness [22], and González-Navarro et al. (2015) identified 59 years as a threshold above which the risk increases [20]. Another recent paper by Orlando and Cruz et al. (2024) found that vertigo and vestibular complications were more common in adults, especially in those over 42 years old, even if often associated with comorbidities such as hypertension, diabetes, renal failure, and depression [60]. Conversely, other studies did not confirm this association: Veroul et al. (2020) found no correlation between age at implantation and postoperative dizziness [56], a result further supported by Forli et al. (2024) [31]. Additional research in elderly populations (80–90+ years) found that while transient balance symptoms were common, the incidence did not differ significantly between age groups, and age was not correlated with persistent vertigo or poorer outcomes [61,62,63].

Those findings support the hypothesis that age could represent more of a contextual modifier than an independent risk factor: postoperative vertigo may be more likely in older patients with associated morbidities, but chronological age alone is not consistently predictive.

#### 4.1.2. Hearing Loss Aetiology

Many aetiologies underlying severe-to-profound sensorineural hearing loss also cause vestibular dysfunction. Epidemiological data suggest that approximately 10–50% of CI candidates exhibit some grade of unilateral or bilateral vestibular impairment on preoperative testing [23,64,65]. Histopathological data support this association: in profound deafness, spiral ganglion neuron loss often parallels a marked reduction in Scarpa’s (vestibular) ganglion cells [27].

Prior ablative treatments or surgical implications should also be considered in some specific aetiologies, as in Ménière’s disease. In this context, a pooled analysis reported that 27% of patients had undergone ablative interventions prior to CI, with 7.4% of intratympanic gentamicin, 10% of endolymphatic sac decompression surgery, 3.4% of labyrinthectomy, and 0.6% vestibular neurectomy [66].

Test-specific abnormalities are variably distributed in different aetiologies, with vHIT abnormal in 8–18%, caloric hypofunction in 19–50%, and absent cVEMP responses in 29–68% before surgery [12]. As discussed below, preoperative VEMPs can even show a high prevalence of otolith dysfunction in adults evaluated for CI, with recent cohorts and reviews reporting abnormal or absent cVEMP and/or oVEMP responses in roughly 30–65% of candidates [28,67], and caloric hypofunction or canal paresis is reported in 37–65% [68,69]. According to Bittar et al. (2019), this aspect makes caloric testing more sensitive than vHIT for detecting vestibular impairment before CI surgery [68].

Furthermore, in a small cohort of patients, Miwa and colleagues (2019) reported that up to 66% of adult CI candidates can show abnormal stabilometry before surgery, especially those with pre-existing vestibular dysfunction [70].

In this context, side selection for CI should integrate ear-specific audiologic and vestibular data, especially in subjects with aetiologies that may evoke vestibular concerns. Audiology aspects should prevail; however, if expected hearing outcomes are comparable, implanting the more vestibular-impaired ear can better preserve the vestibular reserve and avoid the risk of a severe bilateral hypofunction, leading to chronic balance disorders.

#### 4.1.3. Pre- Versus Postoperative State of Vestibular System

Two different meta-analyses indicate that CI surgery can even further affect the vestibular function [28,71], and some studies report that between one-third and two-thirds of patients demonstrate some postoperative deterioration of vestibular function on objective testing [72,73].

After CI, approximately 30% of patients can show an abnormal vHIT, predominantly involving the ipsilateral horizontal canal. Notably, some patients develop new corrective saccades despite gains remaining within normal limits; thus, vHIT might detect subtle high-acceleration changes without overt hypofunction [69]. Anyway, as introduced in the previous paragraph, some authors indicate that low-frequency lateral canal function is even more vulnerable, with caloric abnormalities that increase significantly post-CI. Roughly one-third of adults, indeed, show new or worsened canal hypofunction at caloric testing, while complete areflexia remains rare [39,69,71]. In one clinical series only, caloric testing showed a high rate of postoperative canal hypofunction, with ipsilateral hyporeflexia in 32% and complete areflexia in 10% on the implanted side [74].

But as previously discussed, the most frequent instrumental abnormality seems to concern saccular vestibulo-collic reflexes, with cVEMPs that resulted in attenuated or absent after CI in >85% of cases in some series [22,26]; oVEMPs seemed less affected, indicating that the saccular dysfunction generally exceeds the utricular involvement.

Another confirmation of these aspects can be found in the meta-analysis by Ibrahim et al. (2017), which concluded that the overall clinical impact of CI on vestibular function is modest, with caloric responses and VEMPs that are most often altered post-CI, whereas no consistent effect is observed on vHIT or stabilometry [71]. And even De Castro and colleagues (2022), in another recent meta-analysis, confirmed this aspect, concluding that postoperative abnormal findings were observed mainly on VEMP testing, whereas the other vestibular assessments retained a high proportion of normal results after CI surgery [28].

Overall, then, the literature aligns with clinical experience: in most adults, vestibular symptoms after CI are mild and self-limited, and any subtle postoperative hypofunction is typically compensated over time. Conversely, patients with significant preoperative vestibular hypofunction are at higher risk of postoperative imbalance and should be counselled accordingly, with tailored follow-up and early vestibular rehabilitation when appropriate.

#### 4.1.4. Additional Factors

Additional patient-related modulating factors could be represented by polypharmacy, cardiovascular comorbidities, central nervous system disease, and pre-existing functional balance disorders (such as PPPD). However, the marked heterogeneity of available data precluded a specific analysis, but they are likely to contribute to interindividual phenotypic variability, and targeted investigation in future prospective studies is advisable.

### 4.2. Surgical Determinants

Surgery may influence postoperative vestibular conditions through multiple mechanisms:

- First, surgical trauma can proportionally reduce vestibular reserve on the operated side, precipitating an acute postoperative vestibular syndrome due to unilateral hypofunction and/or contributing to chronic postoperative balance disorders [28,71].
- Second, more traumatic techniques may increase the likelihood of postoperative BPPV by facilitating the ingress of bone dust or blood into the labyrinth and by promoting otoconial dislodgement [19,33,34].
- Third, more aggressive surgery may elicit a stronger inflammatory response, leading to more conspicuous fibrosis and a higher risk of postoperative hydropic events, including secondary endolymphatic hydrops [39,40,41].

Evidence seems to support that the round-window approach can be the preferred technique to minimize vestibular injury during cochlear implantation, with multiple prospective cohorts and comparative studies reporting lower rates of vestibular end-organ dysfunction and fewer postoperative vertigo symptoms with the round-window approach rather than with cochleostomy. In some papers, instrumental testing (especially cVEMP but also caloric and vHIT) seems to detect less vestibular impairment after the round-window approach, and histopathology studies reported reduced trauma and fibrosis [75,76,77]. Some other series, however, find no significant difference between approaches, likely reflecting variation in patient selection, electrode type, and assessment methods [78]. Globally, the heterogeneity across studies warrants cautious interpretation.

### 4.3. Device and Programming Variables

Current studies do not demonstrate a clear influence of device types (including electrode array characteristics, brand, or insertion depth) on postoperative vertigo or vestibular dysfunction. In fact, large retrospective cohorts and comparative studies report no significant association between device characteristics and either short- or long-term vestibular complaints [28,54,55,56,79]. So, at present, device type per se is not a reliable predictor of postoperative vestibular complaints.

On the other hand, electrode misplacement into the vestibule, in a semicircular canal, or the internal auditory canal can lead, quite obviously, to vestibular damage and, in some cases, severe sequelae [80,81,82,83]. In these cases, intraoperative electrophysiological testing (e.g., neural response telemetry [NRT]) can raise an early suspicion through atypical impedances or NRT patterns. Meanwhile, imaging (intraoperative radiography/fluoroscopy; postoperative X-ray or CT) can confirm eventual electrode malposition. Also, cases of delayed identifications of misplacement are reported, particularly when patients present without vestibular symptoms, showing only poor speech performance or unusual non-auditory sensations [80,81,83,84].

These findings support that, in case of difficulties during surgery, subtle abnormalities in neural telemetry or unexpectedly poor implant performance should trigger targeted imaging to exclude malposition, even if vestibular complaints are absent [84,85].

## 5. Conclusions and Future Directions

Vestibular symptoms after cochlear implantation are common but mostly transient and clinically mild. Objective instrumental abnormalities are frequently observed both pre- and postoperatively, but clinical impact is often small. However, possible postoperative vestibular disturbances should be addressed with patients during preoperative counselling.

The post-CI spectrum of vestibular complaints is indeed heterogeneous and includes five main phenotypes: acute postoperative vertigo, BPPV, delayed Ménière-like episodes, chronic disequilibrium, and, very infrequently, stimulation-linked phenomena.

This heterogeneity, together with non-uniform definitions and controversies about laboratory test timing, explains the wide range of incidence estimates across studies. Multiple pathophysiological mechanisms have been proposed for each phenotype, partially supported by instrumental data. Management of postoperative vestibular conditions should then be phenotype-driven.

Patient factors (age, aetiology of deafness, and pre-existing vestibular impairment) and surgical factors (atraumatic technique and approach) plausibly modulate risk, whereas device type per se shows no consistent association with vestibular outcomes. Pre-existing vestibular conditions represent the most reported risk factor for postoperative vestibular complaints. However, in the setting of uneventful surgery, current evidence is weak for any single factor acting as an independent, robust predictor of postoperative vertigo.

Finally, preoperative vestibular function is a factor to consider in CI side selection; when hearing outcomes are comparable, it may be advisable to implant the more vestibular-impaired ear to preserve contralateral reserve and reduce the risk of bilateral severe hypofunction.

Building on a phenotype-based clinical framework, future research should focus on phenotype-specific mechanisms and risk modifiers of vestibular complications after CI. Standardized definitions would allow prospective studies to investigate whether patient-related factors—such as age, aetiology of hearing loss, and preoperative vestibular status—predispose to specific postoperative presentations, rather than vestibular complaints as a generic outcome.

Similarly, surgical and technical variables should be analysed in relation to defined phenotypes, for example, to clarify whether different approaches (round window versus cochleostomy) are differentially associated with acute postoperative vertigo, BPPV, or delayed hydropic symptoms.

From a clinical perspective, such an approach could directly inform preoperative counselling, audiovestibular assessment, side selection, surgical planning, and targeted postoperative management, ultimately improving risk stratification and patient-centered decision-making.

In conclusion, we hope that a phenotype-based, standardized approach may enhance both research consistency and the clinical management of vestibular complaints after CI.

## Data Availability

The original contributions presented in this study are included in the article. Further inquiries can be directed to the corresponding author.

## Supplementary Materials

The following supporting information can be downloaded at https://www.mdpi.com/article/10.3390/audiolres16020050/s1. Table S1. Summary of selected studies reporting postoperative vestibular complaints after cochlear implantation in adults, with classification according to the proposed phenotype-based framework.
