# Supplementary material for: Narrative Review on Vestibular Complaints After Cochlear Implantation in Adults: Defining Heterogeneous Common Symptoms
Source: Audiol Res. 2026 Mar 25;16(2):50. doi: 10.3390/audiolres16020050 (PMC13113645; doi:10.3390/audiolres16020050)
Supplement: Supplementary file 1 [file audiolres-16-00050-s001.zip › audiolres-3909829-supplementary.pdf]

Table S1. Summary of selected studies reporting postoperative vestibular complaints after cochlear implantation in adults, with classification according to the proposed phenotype-based framework.

Article	Number of Patients Included	Type of Vestibular Complaint	Phenotype (Estimated)
Sosna-Duranowska et al. 2020 [8]	101 (40 with symptomatic, 39.6%)	early (within the first 4 weeks after cochlear implantation) = 25 (24.8%)<br>transient (lasting up to 4 weeks, resolved spontaneously) =15/25<br>protracted (lasting 1-6 months) = 8/25<br>persistent (>6 months) =2/25<br><br>late (>4 weeks after cochlear implantation but within 2 years) = 2 (2%)<br><br>preoperative = 13 (12.9%)<br><br>no symptoms = 61 (60%)<br><br>Different types of vestibular complaints, mostly mixed	Acute Postoperative Vertigo = 25/101 (24.8%)<br><br>BPPV–like Vertigo = 4/101 (4%) + 2 preoperative<br><br>Postoperative Ménière-like Delayed Vertigo and Endolymphatic Hydrops = 2/101 (2%)<br><br>Chronic Postoperative Balance Disorders = 2/101 (2%)<br><br>Electrically Induced Vestibular Symptoms = 0/101 (0%)<br><br>Undefined = 7/101 (6.9%)
Krause et al. 2009 [22]	47 (21 symptomatic, 44%)	Directly after CI = 11 (23%)<br><br>1st day to 1st week = 6 (12%)<br><br>1-4 weeks after CI = 2 (4%)<br><br>1-4 months after CI =1 (2%)<br><br>>6 months after CI = 1 (2%)<br><br>No symptoms = 26 (55%)	Acute Postoperative Vertigo = 19/47 (40%)<br><br>BPPV–like Vertigo = not clearly reported<br><br>Postoperative Ménière-like Delayed Vertigo and Endolymphatic Hydrops = 2/47 (4.3%)<br><br>Chronic Postoperative Balance Disorders = 2/47 (4.3%)<br><br>Electrically Induced Vestibular Symptoms = 0/47 (0%)
Rah et al. 2019 [23]	66 (18 sympthomatic, 27.3%)	Immediate, transient = 10 (15%)<br>Recurrent, episodic = 4 (6%)<br>Immediate, prolonged = 4 (6%)<br><br>Transient vestibular paresis = 8 (12%)<br>BPPV = 2 (3%)<br>Endolymphatic hydrops = 3 (4.5%)<br>Vestibular migraine = 1 (1.5%)<br>Bilateral hypofunction = 3 (4.5%)<br>Unknown etiology = 1 (1.5%)	Acute Postoperative Vertigo = 14/66 (21.2%)<br><br>BPPV–like Vertigo = 2/66 (3%)<br><br>Postoperative Ménière-like Delayed Vertigo and Endolymphatic Hydrops = 3/66 (4.5%)<br><br>Chronic Postoperative Balance Disorders = not clearly estimable<br><br>Electrically Induced Vestibular Symptoms = 0/66 (0%)
Zanetti et al. 2007 [19]	62 (4 symptomatic for BPPV only 6.4%)	BPPV = 4 (6.4%, 12.5% of adult recipients)	BPPV–like Vertigo = 4/62 (6.4%)
Limb et al. 2005 [33]	540 (12 symptomatic for BPPV only 2.2%)	BPPV = 12 (2.2% of adult recipients)	BPPV–like Vertigo = 12/540 (2.2%)
Viccaro et al. 2007 [34]	70 (8 symptomatic for BPPV only 11%)	BPPV = 8 (11% of adult recipients)	BPPV–like Vertigo = 8/70 (11%)

Kwok et al. 2024 [39]	21 all symptomatic	Presumed post-CI secondary endolymphatic hydrops = 10(48%)<br>Of which + BPPV = 2 (10%)<br><br>Exacerbation of existing Meniere Disease = 6 (29%)<br>Of which + Vestibular Migrane = 2 (10%)<br><br>Presumed Autoimmune Ear Disease = 2 (10%)<br>Of which + BPPV = 1 (5%)<br><br>Vestibular Migrane = 1 (5%)<br><br>Unknown = 2 (10%)	Acute Postoperative Vertigo = not reported<br><br>BPPV–like Vertigo = 2/21 (10%, associated with hydrops)<br><br>Postoperative Ménière-like Delayed Vertigo and Endolymphatic Hydrops = 16/21 (76.2%)<br><br>Chronic Postoperative Balance Disorders = not reported<br><br>Electrically Induced Vestibular Symptoms = not reported<br><br>Note: the study was focused on patients with post-CI hydropic symptoms.
Coordes et al. 2012 [57]	104 (20 symptomatic for sound-induced vertigo 18%)	Patients' Survey with the Questionnaire for Sound-Induced Vertigo = 20 (18%) postoperatively<br>10 (9%) preoperatively (excluded)	Electrically Induced Vestibular Symptoms = 20 (18%)

Single case reports were excluded. CI, cochlear implantation; BPPV, benign paroxysmal positional vertigo; MD-like/DEH, Ménière-like vertigo or delayed endolymphatic hydrops.
